# Quantum vortex formation in the “rotating bucket” experiment with polariton condensates

**DOI:** 10.1126/sciadv.add1299

**Published:** 2023-01-25

**Authors:** Ivan Gnusov, Stella Harrison, Sergey Alyatkin, Kirill Sitnik, Julian Töpfer, Helgi Sigurdsson, Pavlos Lagoudakis

**Affiliations:** ^1^ Hybrid Photonics Laboratory, Skolkovo Institute of Science and Technology, Territory of Innovation Center Skolkovo, Bolshoy Boulevard 30, building 1, 121205 Moscow, Russia.; ^2^ School of Physics and Astronomy, University of Southampton, Southampton SO17 1BJ, UK.; ^3^ Science Institute, University of Iceland, Dunhagi 3, IS-107 Reykjavik, Iceland.

## Abstract

The appearance of quantized vortices in the classical “rotating bucket” experiments of liquid helium and ultracold dilute gases provides the means for fundamental and comparative studies of different superfluids. Here, we realize the rotating bucket experiment for optically trapped quantum fluid of light based on exciton-polariton Bose-Einstein condensate in semiconductor microcavity. We use the beating note of two frequency-stabilized single-mode lasers to generate an asymmetric time-periodic rotating, nonresonant excitation profile that both injects and stirs the condensate through its interaction with a background exciton reservoir. The pump-induced external rotation of the condensate results in the appearance of a corotating quantized vortex. We investigate the rotation frequency dependence and reveal the range of stirring frequencies (from 1 to 4 GHz) that favors quantized vortex formation. We describe the phenomenology using the generalized Gross-Pitaevskii equation. Our results enable the study of polariton superfluidity on a par with other superfluids, as well as deterministic, all-optical control over structured nonlinear light.

## Teaser

The "rotating bucket" experiment with polariton superfluids, at GHz-frequencies, reveals quantized vortex formation.

## INTRODUCTION

Orbital angular momentum (OAM) in paraxial light, or optical vorticity, is an essential degree of freedom, alongside polarization, for optical information encoding and processing (*1*). This has sparked a strong interest in developing microlasing devices emitting a rotating phase front of the electromagnetic field of controlled OAM (*2*). Being almost strictly noninteracting systems, optical vortices differ markedly from conventional vortices in interacting fluids. The latter are ubiquitous in nature, ranging from the enormous vortex storms in the gas belts of Jupiter (*3*) to tiny micrometer-size quantum vortices in interacting macroscopic quantum systems such as superconductors (*4*), superfluids (*5*), and Bose-Einstein condensates (BECs) (*6*). While optical vortices are geometric in origin, described typically by Laguerre-Gaussian solutions of the paraxial wave equation, vortices in superfluids and BECs are referred to as topological defects with quantized circulation due to the single-valued nature of their wave function.

Exciton-polariton condensates (*7*), appearing in the strong light-matter coupling regime in semiconductor microcavities (*8*), lie at the interface between noninteracting optical systems and interacting quantum fluids. They can form superfluid currents at elevated temperatures (*9*–*11*) and quantum vortices (*12*–*18*) and, because of their strong nonequilibrium nature, populate geometric vortex states based on the balance of pump-induced gain and dissipation in confining potentials (*19*–*26*). The salient features of exciton-polaritons (hereafter polaritons) are the extremely small effective mass due to the photon part and large nonlinearities due to their excitonic component. The lightness of polaritons renders them excellent candidates for fundamental studies and application of polariton condensates at room temperature (*27*–*30*). However, despite the recent progress in the field of polaritonics, to date, vortex formation in a stirred polariton condensate, as in the “rotating bucket” experiments of liquid helium (*5*) or diluted quantum gases (*6*, *31*–*33*), remains elusive predominately because of their ultrashort lifetime, limited typically to a few picoseconds. While resonant injection of OAM into polariton fluids has been possible for some time (*14*, *16*), the concept of dissipative superfluidity holds only under nonresonant excitation (*34*). Generation of polariton vorticity with deterministic direction of rotation could be possible using external electric fields inducing a pseudodrag effect (*35*) or magnetic fields in combination with cavity transverse electric-transverse magnetic (TE-TM) splitting (*36*).

Here, we realize the rotating bucket experiment in a polariton condensate using a cylindrically asymmetric in-plane optical trap induced by a composite nonresonant excitation beam that is used to inject a repulsive exciton reservoir. The excitation pattern is formed by the beating note of two frequency-detuned single-mode lasers of opposite OAM that allows us to realize a dumbbell-shaped trap that rotates at ad hoc frequencies. This composite beam is used to both create and stir the condensate through the effective torque exerted by the asymmetric shape of the photoexcited exciton reservoir. Because of the finite cavity lifetime, polaritons decay from the cavity through quasi-mode coupling to a continuum of photon states, carrying all the physical information of the condensate: density, energy, momentum, spin, and phase. We use spatially resolved interferometry to investigate the formation of topological phase defects in the condensate as a function of rotation frequency and identify the critical conditions under which quantized vortices are formed. We observe the formation of deterministic quantized vortex states that corotate with the excitation pattern for a range of pump rotation frequencies. The frequency is lower-bounded to the extent to which the optically injected exciton reservoir is sufficiently populated along the circumference of the pump’s rotating profile in order for it to confine the corotating condensate. The higher bound of the rotation frequency is also limited by the exciton recombination rate. When the rotation frequency exceeds the exciton recombination rate, the asymmetry of the reservoir is smeared out, resulting in a cylindrical symmetric trapping potential that does not exert any torque to the condensate.

## RESULTS

**Fig. 1.**
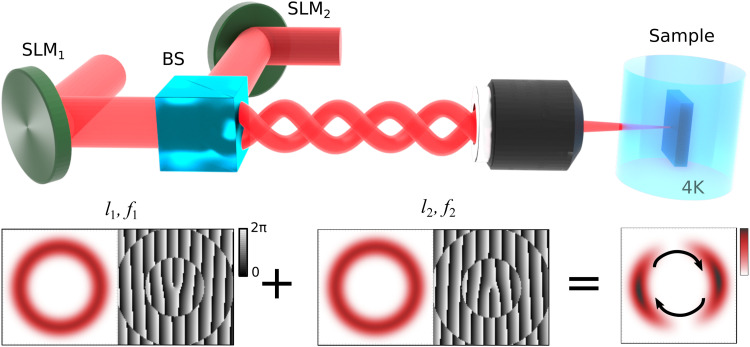
The pumping configuration in the rotating bucket experiment with polaritons. The top part depicts the optical excitation path of the two frequency-detuned, *f*_1_ − *f*_2_ ≠ 0, spatially modulated, single-mode lasers. The bottom part illustrates the spatial intensity profile (in red color) of the two lasers that are individually shaped, with two phase-only spatial light modulators (SLMs), to form ring-like intensity profiles of opposite optical angular momentum *l*_1,2_. The composite excitation beam acts as a rotating dumbbell-shaped polariton trap that both injects and stirs the forming polariton condensate, similar to the optical ferris wheel for ultracold atoms. The grayscale images are the corresponding “perfect” vortex phase masks applied on the SLMs.

We optically inject polariton condensate in a 2λ inorganic microcavity consisting of two strain-relaxed, phosphor-compensated, GaAs/AlAs_0.98_P_0.02_ distributed Bragg reflectors, with InGaAs quantum wells embedded at the antinodes of the intracavity optical field (*37*). The polariton lifetime is τ_p_ ≈ 5 ps, and the exciton-photon detuning is −3.2 meV. The sample is held in a closed-cycle, cold-finger cryostat at 4 K. The excitation beam is composed of two cocircularly polarized, individually wavelength-tunable, frequency-stabilized, single-mode, Ti:sapphire lasers that allow for precise control of their frequency detuning. Their respective wavelengths are centered around the first Bragg minimum of the reflectivity stop-band to minimize reflection losses. In Fig. 1, we schematically depict the excitation part of the experimental setup with the two lasers diffracting from two phase-only reflective spatial light modulators (SLMs). We apply the so-called perfect vortex mask (*38*) on both SLMs to generate annular beam profiles with a tunable beam diameter and a rotating phase front $E_{1,2}\propto e^{i(l_{1,2}\theta -\omega_{1,2}t)}$, where θ is the microcavity in-plane angle and ω_1,2_ are angular frequencies of excitation lasers. Both applied masks are identical except for the relative sign of the OAM. We imprint opposite OAM between the two SLMs; *l*_1_ = ± 1 from SLM_1_ and *l*_2_ = ∓ 1 from SLM_2_. Next, the two spatially modulated lasers are superimposed on a nonpolarizing beam splitter and form a rotating dumbbell-shaped excitation pattern (see Fig. 1). The direction and frequency of the rotation follow from the expression (*39*)

$$f^{\prime }=\frac{\Delta f}{\Delta l}=\frac{f_{1}-f_{2}}{l_{1}-l_{2}}=\frac{1}{2\pi }\frac{\omega_{1}-\omega_{2}}{l_{1}-l_{2}}.$$ (1)

Here, positive and negative $f^{\prime }$ correspond to counterclockwise and clockwise rotation of the intensity pattern, respectively. The nonresonant, composite excitation pattern is projected onto the sample with an average diameter of 14 μm. For zero-frequency detuning between the two lasers, a static (nonrotating) dumbbell-shaped hot exciton reservoir is injected that partially traps polaritons within the excitation profile because of the repulsive interaction between excitons and polaritons. With increasing optical excitation density, we observe the formation of a polariton condensate with its center of mass at the center of the excitation profile, in accordance with previous studies using an annular excitation profile (*40*). Because of the cylindrical asymmetry of the repulsive dumbbell-shaped exciton reservoir, here, the condensate is also spatially asymmetric as previously observed for elliptical pumping profiles (*41*).

**Fig. 2.**
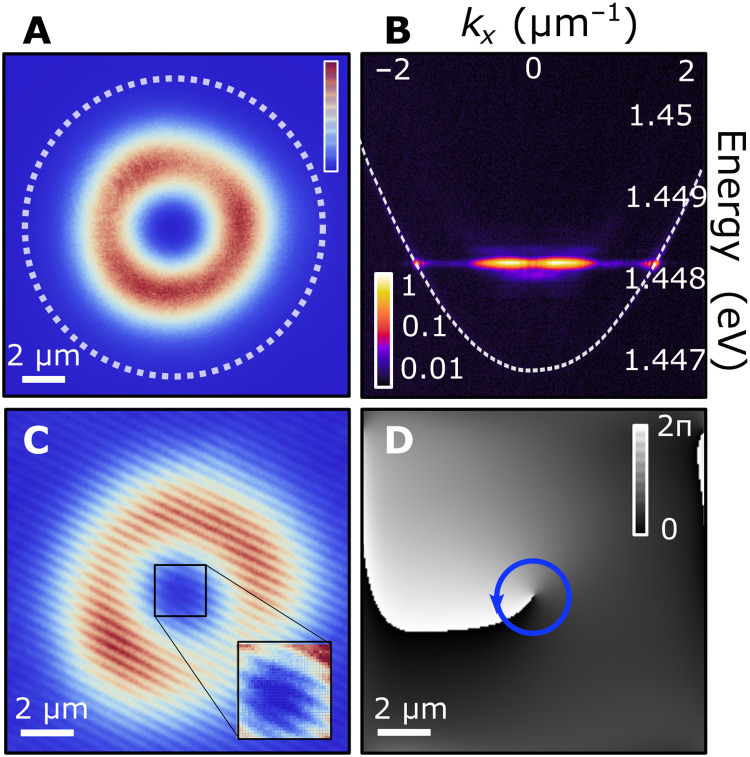
Quantized vortex formation in the rotating bucket experiment. (**A**) Real-space normalized photoluminescence intensity of a polariton condensate under a nonresonant, counterclockwise rotating excitation beam [Δ*f* = 4.6 GHz, (*l*_1_, *l*_2_) = (1, − 1)]. The dashed line corresponds to the circumference of the effective optical trap. (**B**) Angularly resolved normalized photoluminescence intensity of the trapped condensate (false color in logarithmic scale). The white dashed curve depicts the lower polariton dispersion branch. (**C**) Interference pattern of the condensate emission with a resonant, plane-wave, reference laser, revealing a fork-like dislocation in the center of the condensate wave function (see magnified region of interest). (**D**) Phase distribution of the condensate wave function showing a counterclockwise winding phase singularity confirming the formation of a quantized vortex (in false gray scale).

To investigate the effect of stirring a polariton condensate by rotating the excitation pumping profile, we apply a counterclockwise rotating excitation pattern, with positive detuning between the two lasers, Δ*f* = 4.6 GHz, opposite OAM, (*l*_1_, *l*_2_) = (1, − 1), and pumping power of *P* = 1.1*P*_th_, where *P*_th_ is the condensation threshold pump power. Figure 2A shows time-integrated, spatially resolved, photoluminescence measurements, wherein the dotted white circle depicts the circumference of the optical trap, containing an annular-shaped condensate with an intensity minimum in the center of the pumping area. We spectrally resolve the emission in reciprocal space and observe the monochromatic characteristic of a trapped condensate (see Fig. 2B) (*42*). To measure the real-space phase map of the condensate, we apply homodyne interferometry using a resonant plane reference wave (*43*). Figure 2C shows the resulting interference pattern that reveals a fork-like dislocation in the center of the annular-shaped emission pattern, implying a phase singularity. Next, we perform off-axis digital holography technique to extract the real-space phase pattern presented in Fig. 2D. In the center of the condensate wave function, at the minimum of the intensity distribution, we obtain a singularity that is indicative of a quantized vortex, with the counterclockwise phase winding around the vortex core with OAM *l* = 1, corotating with the excitation pumping profile. We note here that, for a stationary annular trap of the same diameter, we do not observe the formation of a vortex state of definite charge (*40*, *42*).

Our results are qualitatively reproduced through numerical modeling using a generalized two-dimensional (2D) Gross-Pitaevskii equation describing the condensate order parameter coupled to an exciton reservoir (see Materials and Methods and section S1). While both co- and counterrotating geometric vortex states are solutions of the effective trapping potential generated by the excitation pumping profile, competition between gain and losses results in a quantum vortex corotating with the exciton reservoir. To unravel the parameter space that defines the prevalence of the corotating configuration, we have derived a reduced generalized Gross-Pitaevskii model or, analogously, a nonlinear Rabi flopping model in the rotating wave approximation describing the time-periodic driven dynamics of the two *l* = ± 1 angular harmonics in the rotating potential (see Materials and Methods and section S3). The results are reminiscent of the AC Stark effect, whereas, in our case, an asymmetric Mollow triplet appears because of losses and gain that, alongside repulsive polariton interactions, give precedence to a condensate corotating with the pump.

**Fig. 3.**
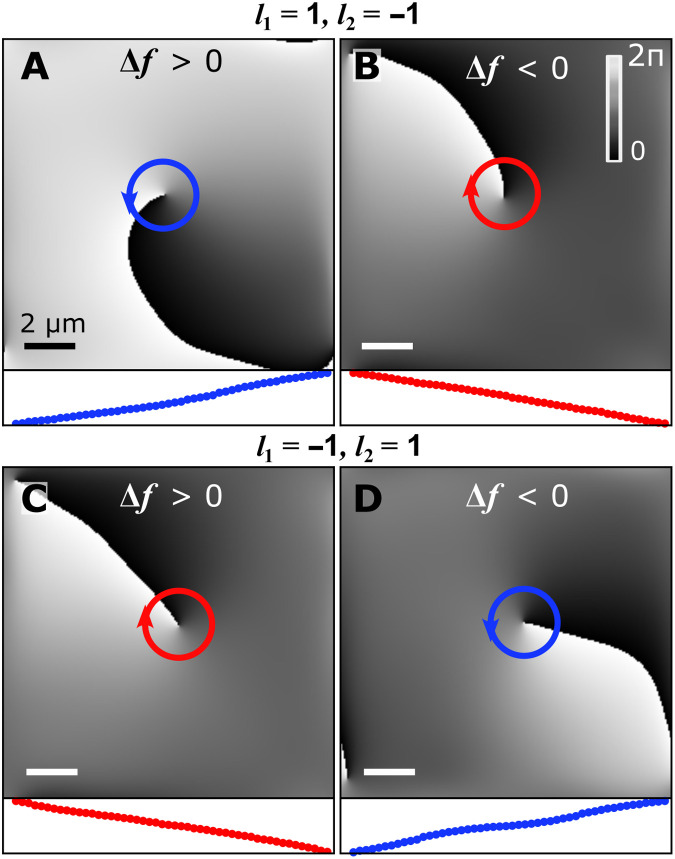
Deterministic control of the quantum vortex charge. Real-space phase distribution of the condensate wave function demonstrating corotation of the winding of the vortex phase with the excitation beam. (**A** and **D**) A counterclockwise and (**B** and **C**) a clockwise winding of the phase following the excitation beam. The winding of the phase is determined by controlling the OAM *l*_1,2_ and lasers frequency difference Δ*f*. In (A) and (C), Δ*f* = 4.6 GHz, and, in (B) and (D), Δ*f* = − 3.7 GHz. The bottom inset of each panel (red/blue solid dots) show the nearly constant angular phase gradient around the line-profiles of the respective phase singularity.

Further on, we demonstrate deterministic control over the charge of the forming quantum vortex by applying the four different configurations arising from Eq. 1. To control the sign of the rotation of the excitation beam, $f^{\prime }$, we tune either the sign of the difference of OAM, Δ*l*, or the sign of the frequency detuning, Δ*f*. The real-space phase distribution in the four configurations is shown in Fig. 3. The phase profiles presented in Fig. 3 (A and B) correspond to Δ*l* = 2 and those in Fig. 3 (C and D) correspond to Δ*l* = − 2. Figure 3 (A and C) corresponds to positive detuning, Δ*f* = 4.6 GHz, and Fig. 3 (B and D) corresponds to negative detuning, Δ*f* = − 3.7 GHz. In all four configurations, we observe the formation of a quantum vortex, with a phase singularity at the core of the vortex and a winding of the phase codirected with the rotation of the excitation beam with nearly constant angular phase gradient; see circular line profiles of the phase in respective lower panels in Fig. 3. Beyond the interest from a fundamental studies perspective on the reproducibility of the formation of quantum vortices in rotating polariton fluids, demonstration of structured nonlinear light sources with controllable topological charge offers applications in classic and quantum communications (*44*–*46*).

**Fig. 4.**
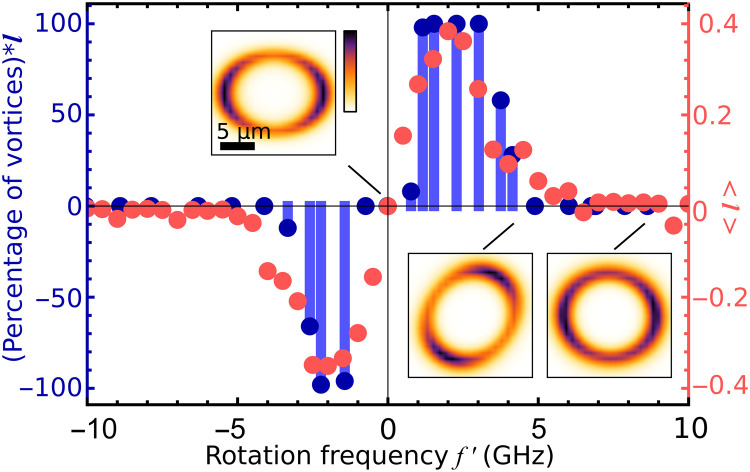
Rotation frequency dependence of a quantum vortex state formation. Histogram of realizations of quantum vortices for *l*_1_ = 1 and *l*_2_ = − 1. Blue markers show the product of the percentage of realizations wherein a quantum vortex occurs multiplied by the optical angular momentum of the resulting state at each rotation frequency $f^{\prime }$. Red markers show the average angular momentum of the confined quantum vortex states obtained using a 2D generalized Gross-Pitaevskii theory. The insets depict snapshots of the exciton reservoir density distribution for 0-, 5-, and 10-GHz rotation frequencies.

Of fundamental interest to the dynamics of the rotating bucket experiment in polariton condensates is the rotation frequency dependence in the formation of quantum vortices. Tuning the rotation frequency of the excitation pattern with a diameter of 14 μm, we observe quantum vortex formation between ±1 and ±4 GHz. Because of the stochastic nature of vortex formation at the edges of this frequency range, we collect a large sample of realizations (>10^3^) for rotation frequencies $f^{\prime }$ spanning from −10 to 10 GHz at a constant (above condensation threshold) excitation density of the composite rotating beam. For each frequency, we record the interference of the real-space condensate photoluminescence with its retroreflected, shifted image and extract the real-space phase distribution for 100 “single-shot” realizations, each, time-integrated over a 10-μs-long excitation pulse width (see section S2). To distinguish quantum vortex states from other states in this large statistical sample, we develop a vortex sorting algorithm (see section S2). Condensate realizations that do not qualify as vortex states in the experiment correspond typically to three scenarios: a condensate populating a Gaussian-like ground state, a condensate fractured across multiple distinct energy state, or a mixture of OAM *l* = ± 1 forming a standing wave (dipole state). These cases would result in flat, indeterminate, or step-like real-space condensate phase profiles, respectively. With blue bars in Fig. 4, we plot the product of the number of instances, where a quantum vortex is observed, and the OAM of the corresponding vortex state. In agreement with the results presented in Fig. 3, the flipping of the condensate vortex charge with the direction of rotation is evidenced in Fig. 4, where the distribution, evidently, inverts around $f^{\prime }=0$.

For small rotation frequencies, $f^{\prime }$ ∈ [ − 1,1] GHz, due to the finite exciton recombination time, the exciton reservoir induced by the dumbbell-shaped excitation pattern is not sufficiently populated to build a confining repulsive potential for a quantum vortex to form, as is the case for a static excitation beam. For rotation frequencies in the range of ±1 and ±4 GHz, respectively, we observe that the formation of a quantum vortex state becomes statistically significant, reaching nearly 100% occurrence approximately at ±2.5 GHz. The resonant-like occurrence of quantized vorticity, for this range of frequencies, suggests that the repulsive potential from the optically induced exciton reservoir can host an excited geometric vortex state above the trap ground state, as shown in the dispersion image of Fig. 2B, and the effective stirring starts inducing a definite direction of vortex rotation. For rotation frequencies ∣$f^{\prime }$∣ higher than 4 GHz, the asymmetry of the reservoir is smeared out, resulting in a cylindrical symmetric trapping potential that does not exert sufficient torque to the condensate for inducing the formation of a quantum vortex state. Condensate in the case occupies the Gaussian ground state (see section S2). The insets in Fig. 4 show snapshots of the modeled exciton reservoir density calculated for three different rotation frequencies, with the highest rotation frequency resulting in an almost circular profile (see Materials and Methods). In the current excitation configuration, the forging of a cylindrical symmetric trapping potential at higher frequencies prevents the formation of a higher number of quantum vortices with increasing rotation frequency, as it was observed in other superfluid systems. The experimental observations are qualitatively confirmed through numerical simulations of the 2D generalized Gross-Pitaevskii model coupled to an exciton reservoir (see Materials and Methods and section S1). The numerical results for the average OAM of the confined quantum vortices as a function of rotation frequency are shown with red solid markers in Fig. 4.

## DISCUSSION

The investigation of quantum vortex formation in ultracold trapped quantum gases and liquid helium has enabled a plethora of fascinating fundamental and comparative studies of superfluids. Here, we realize and study the formation of quantum vortex states in rotating bucket experiment of driven-dissipative quantum fluids of light based on BECs of polaritons. We emphasize that, similarly to the classical rotating bucket experiments (*5*, *6*), obtained rotation frequency dependence for our system manifests both critical frequency (velocity) at which vortex appears ($f^{\prime }$ = 1 GHz) and the range of stirring frequencies that sustains vortex of deterministic topological charge. However, the underlying physics of polaritons shifts required stirring frequencies to gigahertz range (compared to sub-hertz for the superfluids and BECs) and does not support the transition to higher topological charges. Beyond facilitating means for the fundamental study of quantum fluids of light and comparative studies with other superfluids, polariton condensates offer an alternative versatile semiconductor platform for generating nonlinear structured light. With the rapid advancements in creating extended polariton networks (*43*), our method can be used to engineer vortex arrays to study the complex interplay of polarization, OAM, and linear momentum degrees of freedom in programmable large-scale driven-dissipative quantum fluids. Moreover, our demonstration offers a controllable source of optical vortices that empower applications in classical (*47*–*49*) and quantum computing (*50*, *51*) and the potential to study nonreciprocal transport of quantum fluids (*52*).

## MATERIALS AND METHODS

### Generalized Gross-Pitaevskii theory

The effects of the rotating pump profile on the condensate and exciton reservoirs are modeled using the mean-field theory approach, where the condensate order parameter Ψ(**r**, *t*) describes the condensate density as a macroscopic 2D wave function following the generalized Gross-Pitaevskii equation coupled to an active and inactive exciton reservoirs *n*_A,I_(**r**, *t*) (*53*). The active reservoir excitons undergo bosonic scattering into the condensate, whereas the inactive high-momentum reservoir excitons are not allowed to scatter into it

$$i\hbar \frac{\partial \Psi }{\partial t}=\left[-\frac{\hbar^{2}\nabla^{2}}{2m}+G(n_{A}+n_{I})+\alpha {|\Psi |}^{2}+\frac{i\hbar }{2}(Rn_{A}-\gamma )\right]\Psi$$ (2)

$$\frac{\partial n_{A}}{\partial t}=-(\Gamma_{A}+R{|\Psi |}^{2})n_{A}+Wn_{I}$$ (3)

$$\frac{\partial n_{I}}{\partial t}=-(\Gamma_{I}+W)n_{I}+P(r,t)$$ (4)

Here, *m* is the effective polariton mass, and *G* = 2*g*∣*X*∣^2^ and α = *g*∣*X*∣^4^ are the polariton-reservoir and polariton-polariton interaction strengths, respectively, where *g* is the exciton-exciton dipole interaction strength and ∣*X*∣^2^ is the excitonic Hopfield coefficient. In addition, *R* is the rate of stimulated scattering of polaritons into the condensate from the active reservoir, γ = 1/τ_p_ is the polariton decay rate (inverse of polariton lifetime), Γ_A, I_ are the active and inactive reservoir exciton decay rates, *W* is the inactive to active reservoir exciton conversion rate, and *P*(**r**, *t*) describes the nonresonant continuous-wave dynamic pumping profile

$$P(r,t)=P(r){|e^{i(l_{1}\theta -\omega_{1}t)}+e^{i(l_{2}\theta -\omega_{2}t)}|}^{2}=4P(r)\cos^{2}\left[\frac{(l_{1}-l_{2})\theta }{2}-\frac{(\omega_{1}-\omega_{2})t}{2}\right]$$ (5)

where P(r) represents the annular intensity profiles shown in Fig. 1.

To understand the reservoir dynamics that play an essential role in our vortex stirring experiment, it is good to write the general solution to Eq. 4

$$n_{I}(r,t)=e^{-(W+\Gamma_{I})t}[4P(r)I(\theta ,t)+n_{I}(r,0)]$$ (6)

where the integral becomes

$$\begin{array}{rl} & I(\theta ,t)=e^{(W+\Gamma_{I})\tau }\\ & \;\times \frac{(W+\Gamma_{I})\cos^{2}(\bar{\theta }-\bar{\omega }\tau )-\bar{\omega }\sin[2(\bar{\theta }-\bar{\omega }\tau )]+\frac{2{\bar{\omega }}^{2}}{W+\Gamma_{I}}}{{\left(W+\Gamma_{I}\right)}^{2}+4{\bar{\omega }}^{2}}{|}_{0}^{t} \end{array}$$ (7)

Here, we have simplified the notation to $\bar{\theta }=(l_{1}-l_{2})\theta /2$, and $\bar{\omega }=(\omega_{1}-\omega_{2})/2$. In the limit of a slowly rotating trap at long times one retrieves a reservoir that adiabatically follows the shape of the pump

$${n_{I}(r,t)|}_{W+\Gamma_{I}\gg f\prime }\approx \frac{P(r,t)}{W+\Gamma_{I}}$$ (8)

The same argument can be applied to the active reservoir *n*_A_(**r**, *t*). Assuming that nonlinear effects are weak and that the decay rate Γ_A_ of the active reservoir is fast, i.e., Γ*_A_* ≫ $f^{\prime }$, *R*∣Ψ∣^2^, then it will also adiabatically follow the dynamics of the inactive reservoir

$${n_{A}(r,t)|}_{\Gamma_{A}\gg f\prime }\approx \frac{Wn_{I}(r,t)}{\Gamma_{A}}=\frac{P(r,t)}{\Gamma_{A}(1+\Gamma_{I}/W)}$$ (9)

In the opposite limit of a rapidly rotating trap (*W* + Γ*_I_* ≪ $f^{\prime }$), the inactive reservoir approaches the cylindrically symmetric time-independent solution *n_I_*(**r**) ≈ 2P(*r*)/(*W* + Γ*_I_*). The same goes as well for the active reservoir.

Equations 2 and 3 are numerically integrated in time using a linear multistep method starting always from weak random initial conditions. The parameters used in these simulations are based on the sample properties (*37*), with *m* = 5.3 × 10^−5^*m*_0_, where *m*_0_ is the free electron mass, $\gamma =\frac{1}{5.5}$ ps ^−1^, *g* = 1 μeV μm^2^, and ∣*X*∣^2^ = 0.35. We take Γ*_A_* = γ because of the fast thermalization to the exciton background and the nonradiative recombination rate of inactive reservoir excitons to be much smaller than the condensate decay rate with Γ*_I_* = 0.01γ. The remaining parameters are enumerated through fitting to experimental data, giving *R* = 0.01 ps ^−1^ and *W* = 0.05 ps ^−1^. The nonresonant pump drive term *P*(**r**, *t*) uses a similar profile as in experiment.

For each rotation frequency, $f^{\prime }$, 40 unique realizations are performed, each starting with different random initial conditions and integrated forward in time until converging to a final state. The expectation value of the OAM in the condensate plotted in Fig. 4 is written as

$$\langle l\rangle =\frac{1}{\hbar }\frac{\left\langle \Psi |{\hat{L}}_{z}|\Psi \right\rangle }{\left\langle \Psi |\Psi \right\rangle }$$ (10)

where ${\hat{L}}_{z}$ is the angular momentum operator.

### Perturbative treatment in the linear regime

In this section, we consider a truncated Hilbert space composed of just the *l* = ± 1 OAM modes describing the trapped polaritons in the linear regime under a time-periodic perturbation. The Schrödinger equation describing the driven system without cavity losses γ can be written

$$i\hbar \frac{\partial \psi }{\partial t}=[{\hat{H}}_{0}+\hat{U}(t)]\psi$$ (11)

where

$${\hat{H}}_{0}=-\frac{\hbar^{2}\nabla^{2}}{2m}+\frac{1}{2}m\omega_{∥}^{2}r^{2}$$ (12)

$$\hat{U}(t)=\hbar \lambda \cos^{2}\left(\frac{(l_{1}-l_{2})\theta }{2}-\frac{(\omega_{1}-\omega_{2})t}{2}\right)$$ (13)

Here, we have assumed that the polaritons are deeply enough trapped to feel an effective harmonic potential of strength ω_∥_ determined by the time-independent terms in the reservoir solutions (Eqs. 6 to 9). Here, λ = λ*_R_* + *i*λ*_I_* is a complex number that satisfies ∣λ∣ ≪ ω_∥_ (i.e., the operator $\hat{U}$ can be treated as a perturbation). This is a valid assumption for intermediate rotation frequencies when (ω_1_ − ω_2_)^2^ > 2(*W* + Γ*_I_*)^2^, where the reservoir becomes sufficiently smeared out (see Eqs. 6 and 7), so it forms approximately a sum of a time-independent confinement term $m\omega_{∥}^{2}r^{2}/2$ and a weak time-dependent perturbating term $\hat{U}(t)$.

For ${\hat{H}}_{0}$, the solutions are 2D Hermite- or Laguerre-Gaussian modes (*54*). We will truncate our Hilbert space around the degenerate pair of first excited angular harmonics *l* = ± 1 that are the main observation in this experiment

$$\psi =\xi (r)(c_{+}e^{i\theta }+c_{-}e^{-i\theta })e^{-i2\omega_{∥}t}$$ (14)

where $\xi (r)=\frac{\beta^{2}}{\sqrt{\pi }}re^{-\beta^{2}r^{2}/2}$ is the radial solution to the unperturbed Schrödinger equation with $\beta =\sqrt{m\omega_{∥}/\hbar }$.

The angular dependence of $\hat{U}$ means that it couples harmonics that differ by *l*_1_ − *l*_2_ in angular momentum. Therefore, the case of interest *l*_1_ − *l*_2_ = ± 2 will couple together the harmonics *e*^±*i*θ^. It also means that the ground state is decoupled from the first excited state and therefore is safe to neglect in the expansion (Eq. 14). Plugging Eq. 14 into the perturbed Schrödinger equation (Eq. 11) and integrating over the spatial degrees of freedom (i.e., exploiting their orthogonality), we obtain the coupled system of equations (up to an overall energy factor)

$$i\frac{\partial c_{\pm }}{\partial t}=\frac{\lambda }{4}c_{\mp }\exp\left[\mp i(\omega_{1}-\omega_{2})t\frac{l_{1}-l_{2}}{|l_{1}-l_{2}|}\right]$$ (15)

These are the same equations that describe Rabi flopping in a degenerate two-level system in the rotating wave approximation. This is analogous to the AC Stark effect, commonly applied in atomic optics where an oscillating electric field shifts atomic transitions. The solutions are

$$c_{\pm }(t)=A_{\pm }e^{-i(\pm \Delta \omega +\Omega_{R})t/2}+B_{\pm }e^{-i(\pm \Delta \omega -\Omega_{R})t/2}$$ (16)

where *A*_±_ and *B*_±_ are determined by initial conditions and parameters of the model, Δω = (ω_1_ − ω_2_)(*l*_1_ − *l*_2_)/ ∣ *l*_1_ − *l*_2_∣ with Δω < 0 corresponding to clockwise and Δω > 0 counterclockwise pump rotation, and $\Omega_{R}=\sqrt{\Delta \omega^{2}+{\left(\lambda /2\right)}^{2}}$. We note that the exponents constitute the famous Mollow triplet, the hallmark of dressed quantum states.

We can derive a useful expression that quantifies the amount of OAM in the non-Hermitian system at time scales t≫Im(ΩR−1)=ν−1. It is instructive to describe our initial condition as a vector on the unit sphere with polar and azimuthal angles Θ and ϕ, so it reads $|\psi (t=0)\rangle ={\left(c_{+}^{\left(0\right)},c_{-}^{\left(0\right)}\right)}^{T}={\left(\cos(\Theta /2)e^{i\phi /2},\sin(\Theta /2)e^{-i\phi /2}\right)}^{T}$. We then obtain

∣c+(t)∣2−∣c−(t)∣2≃12∣ΩR∣2[(Δω2−∣λ∣24+∣ΩR∣2)cos⁡(Θ)cosh⁡(νt)+(Im(λΩR∗)sin⁡(ϕ)sin⁡(Θ)−2Re(ΩR)Δω)sinh⁡(νt)−Re(λ)cos⁡(ϕ)Δωsin⁡(Θ)cosh⁡(νt)] (17)

This OAM measure is similar to the Stokes parameter for circularly polarized light, except now for angular momentum. Note that when nonlinearities are included, the divergence is avoided through the reservoir gain clamping down.

Averaging over all the possible initial conditions, Θ = [0, π] and ϕ ∈ [0, 2π), which physically represents averaging over multiple realizations of the condensate seed, we obtain

⟨∣c+(t)∣2−∣c−(t)∣2⟩Θ,ϕ≃−Re(ΩR−1)Δωsinh⁡(νt) (18)

This expression is interesting, as it shows that without ν = Im (Ω*_R_*), the OAM would vanish. Moreover, the amplitude and sign of the OAM is dictated not just by the strength and orientation of the pump Δω but also the anti-Hermitian part *ν* (because the hyperbolic sine is an odd function).

Our experimental observations imply that we must have *ν* < 0 in order for the condensate to be corotating with the pump. This means that our perturbation parameter should satisfy sgn(λ*_R_*λ*_I_*) = − 1, which physically means that the lower-energy components in our Rabi model (*15*) have more gain. Intuitively, this makes sense because polaritons are highly interactive and relax efficiently in energy, preferentially populating low-energy solutions. In section S3, we verify this interpretation by introducing standard polariton condensate nonlinearities to Eq. 15 and numerically solving the coupled equations of motion, averaging over many random initial conditions, for the *c*_±_(*t*) modes in the condensate.

In the special case of *ν* = 0, we get particularly simple expression in the time-average over one period *T_R_* = 2π/Ω*_R_*

$${\left\langle {|c_{+}(t)|}^{2}-{|c_{-}(t)|}^{2}\right\rangle }_{T_{R}}≃\frac{\Delta \omega^{2}}{\Omega_{R}^{2}}\left[\cos(\Theta )-\frac{\lambda }{2\Delta \omega }\cos(\phi )\sin(\Theta )\right]$$ (19)

Notice that if one averages the OAM also over all initial conditions on the unit sphere, then the above expression is zero. This underlines the crucial difference in the OAM dynamics between the Hermitian (*ν* = 0) and non-Hermitian (*ν* ≠ 0) case. [Postsubmission note: the following manuscript was submitted to the arXiv describing an alternative technique for the nonresonant rotation of a polariton condensate (*55*).]

## Supplementary Materials

### This PDF file includes:

Supplementary Text (Sections S1 to S3) Figs. S1 to S3

## Notes

View/request a protocol for this paper from *Bio-protocol*.
