# Supplementary material for: Quantum vortex formation in the “rotating bucket” experiment with polariton condensates
Source: Sci Adv. 2023 Jan 25;9(4):eadd1299. doi: 10.1126/sciadv.add1299 (PMC9876539; doi:10.1126/sciadv.add1299)
Supplement: Supplementary file 1 — Supplementary Text (Sections S1 to S3) Figs. S1 to S3 [file sciadv.add1299_sm.pdf]

Science Advances
AAAS

Supplementary Materials for

Quantum vortex formation in the "rotating bucket" experiment with polariton condensates

Ivan Gnusov *et al.*

Corresponding author: Pavlos Lagoudakis, pavlos.lagoudakis@soton.ac.uk

Sci. Adv. **9**, eadd1299 (2023)
DOI: 10.1126/sciadv.add1299

This PDF file includes:

Supplementary Text (Sections S1 to S3)
Figs. S1 to S3

S1. CONDENSATE INTENSITY AND PHASE DISTRIBUTION SIMULATED WITH 2D GENERALISED GROSS-PITAEVSKII EQUATION.

Our simulations using the generalized Gross-Pitaevskii theory (presented in main manuscript) reveal that the resulting condensate, driven by the rotating excitation profile, forms a similar time-averaged annular density structure (presented in Fig. S1A) to the experimental findings shown in the main text (Figure 2), while possessing the vortex phase singularity (see Fig. S1B) and phase winding co-rotating with the excitation pumping profile.

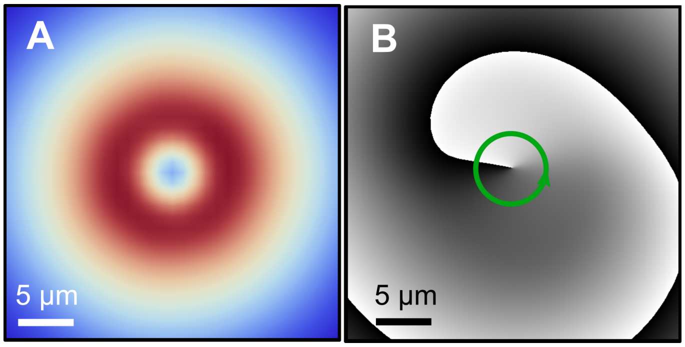

Figure S1. **Condensate intensity and phase distribution simulated with 2D generalised Gross-Pitaevskii equation.** Simulated time integrated real-space intensity **A** and instantaneous phase **B** of the condensate at $f' = 2$ GHz demonstrating the formation of the quantised vortex after 800 ps of numerical integration.

S2. POLARITON INTERFEROMETRY AND VORTEX SORTING PROCEDURE

We retrieve the phase of the polariton condensate utilizing two interferometric techniques: the homodyne interferometry technique [41], and the more common interference of the condensate emission with a retro-reflected copy of itself in a Michelson interferometer [12].

The homodyne technique is described in detail in [41]. We use an external diode laser with the same energy as the condensate as a reference wave and interfere it with the condensate emission. The reference laser beam is greatly expanded to spatially overlap with the entire collected photoluminescence pattern in order to have a flat phase front in the vicinity of the condensate. To lock the phases between the reference laser and the condensate, we use a small portion of the reference laser to locally seed the condensate phase. This allows for the observation of high-contrast interference fringes (see Fig. 2C in the main text) and for the phase retrieval of the condensate.

For the interference measurements of the condensate with itself used to obtain the data presented in Fig. 4 in the main text, we do not use an external light source, but instead we spatially displace one arm of the interferometer with respect to the other (retroreflected) to observe the phase dislocation. As a result, when a vortex is present in the condensate, we see instead a vortex-antivortex pair in the interference pattern due to the angular momentum flipping caused by the retroreflection (see Fig. S2).

Given the interference pattern, one can extract the phase of the condensate wave function by performing the 2D Fourier transformation. In the frequency domain, we filter the harmonic corresponding to the fringes period and do the inverse Fourier transform. The resultant complex-valued array carries the information about the phase of the initial distribution, which we can retrieve by taking the complex argument of the values in this array.

Example of interference images, used to obtain the data in Fig. 4 in the main manuscript, are presented in Figs. S2A,B. Figure S2A corresponds to the $f' = -2.5$ GHz rotation frequency and depicts the forklike dislocations corresponding to the phase singularity of the condensate vortex. Note the two counter-directed forks in the interference pattern Fig. S2A, one of which directly corresponds to the vortex present in the condensate and the other to the spatially shifted and retroreflected condensate signal with flipped optical angular momentum (OAM). Figure S2B corresponds to the condensate occupying the Gaussian ground state of the trap at high rotation frequency $f' = -8.2$ GHz. The corresponding phase profile reveals a flat phase shown in Fig. S2G with no OAM.

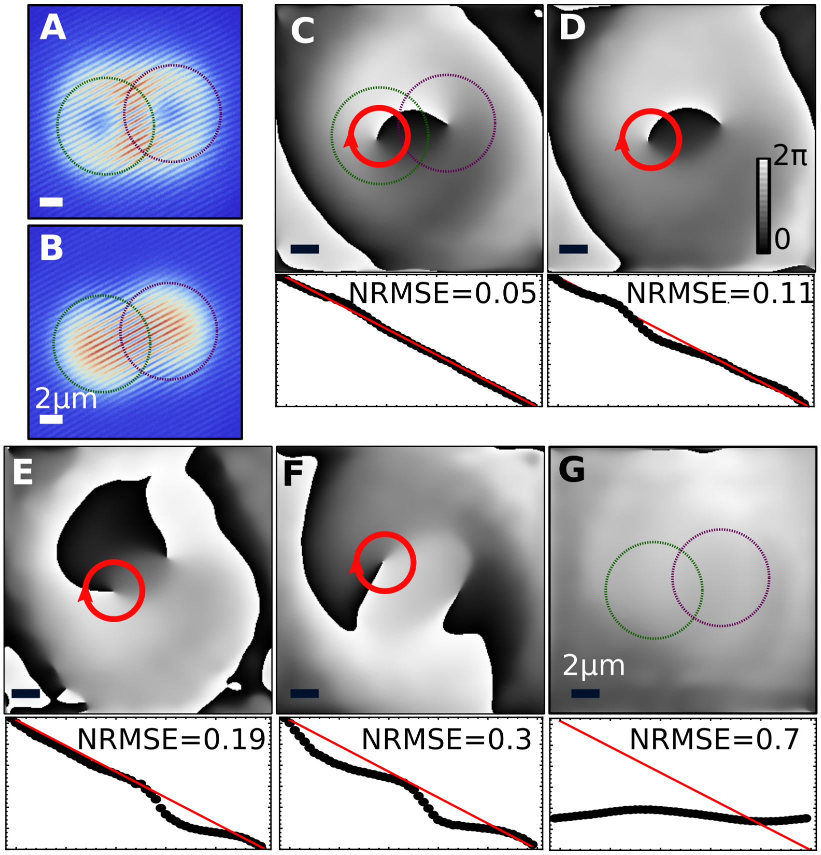

Figure S2. **Examples of polariton condensate phase distribution under different stirring frequencies.** Condensate emission (green ring) interfered with the retro-reflected and displaced copy of itself (purple ring) at $f' = -2.5$ GHz (**A**) and $f' = -8.2$ GHz (**B**). Note, that green and purple circles in (**A**-**C**) schematically depict the maximum of the condensate intensity distribution in Figure a for direct and retro-reflected copy respectively. The less bright and out-flowing part of the condensate is also present and results into the interference fringes outside the rings. (**C**-**G**) Examples of the condensate phase distribution for different realizations with a 10 μs excitation pulse width. The bottom panels of (**C**-**G**) represent the annular line profile with a 2.6 μm radius around each phase singularity unwound starting from the black-white colour dislocation and compared to the phase profile of a perfect antivortex (red line). In panels (**C** and **D**) $f' = -1.5$ GHz, and in (**E** and **F**) $f' = -3.8$ GHz, and in (**G**) $f' = -8.2$ GHz.

Figures S2C-F depict the phase distribution for different condensate realizations at different rotation frequencies (Fig. S2C,D and Fig. S2E,F correspond to $f' = -1.5$ GHz and $f' = -3.8$ GHz, respectively). The circular line profiles around the phase singularities are presented in the lower panels. The difference between the ideal antivortex profile (see the red line) and the experimental phase profile (black dots) appears due to various reasons. First, overlapping the condensate to itself leads to the interference of two anisotropic (non-flat) phase fronts resulting in phase scrambling. Second, the condensate can simultaneously occupy neighbouring energy states (ground and excited states of the trap) which can lead to a mixture of phases corresponding to specific energy states during phase retrieval procedure. The difference between ideal and measured winding in the angular phase profile defines a true/false threshold on whether

a vortex exists in the condensate or not.

Vortex sorting algorithm. For the histogram presented in Fig. 4 in the main manuscript, we take the threshold value of the normalized root mean square error (NRMSE) to the ideal vortex as ≤ 0.2 when defining whether we observe a vortex or not. See, for example, Fig. S2E for typical phase distribution with extracted value of NRMSE $= 0.19$ close to the threshold error value. Extracted value NRMSE $= 1$ means maximum error where all phase datapoints deviate by π radians from the expected profile.

S3. SOLUTION OF REDUCED GENERALIZED GROSS-PITAEVSKII EQUATION

As an extension to our theoretical analysis presented in the main manuscript, we write a reduced generalised Gross-Pitaevskii model by introducing the standard polariton condensate nonlinearities to Eq. (15),

$$i\frac{dc_\pm}{dt} = \left[ip + (\tilde{\alpha} - i)(|c_\pm|^2 + 2|c_\mp|^2)\right] c_\pm + (1 + i\eta)c_\mp e^{\mp i\Delta\omega t}. \tag{S1}$$

As discussed around Eq. (14) in the main text, $c_\pm = \sqrt{N_\pm}e^{i\phi_\pm}$ are complex order parametersr that describe the amount of polaritons in each OAM $l = \pm1$ modes and their phase. Here, p is a denotes the difference between the pump gain and linear polariton losses, $\tilde{\alpha}$ describes the renormalized nonlinear energy shift after integrating out the spatial degrees of freedom. We have also scaled time in units of conservative coupling strength $t \to t/\lambda_R$ and defined $\eta = \lambda_I/\lambda_R$. Note that from our analysis in the main manuscript, we expect a condensate solution co-rotating with the pump when $\text{sgn}(\nu) = \text{sgn}(\eta) = -1$. The results on the time-average $\langle \dots \rangle$ behaviour of the coupled system (in the long time limit) are presented in Fig. S3, also averaged over many random initial conditions, which show that depending on the sign of the dissipative coupling strength η the vorticity can flip with respect to the pump. Here we have set $\tilde{\alpha} = 1$ and $\Delta\omega = 1$ which corresponds to repulsive polariton interactions and counterclockwise rotating pump pattern, respectively. If we flip the sign of $\Delta\omega$ then the colorscale in Fig. S3B also flips. Note the slight bending of the boundary separating blue and red regions which can be attributed to the nonlinear energy term in Eq. (S1) becoming comparable to the dissipative coupling rate η, favoring a co-rotating polariton fluid. If the sign of $\tilde{\alpha}$ is reversed to become negative (i.e., focusing Kerr effect) the boundary will bend the other way. These results evidence that the repulsive polariton interactions favor a co-rotating polariton fluid like we observe in our experiment.

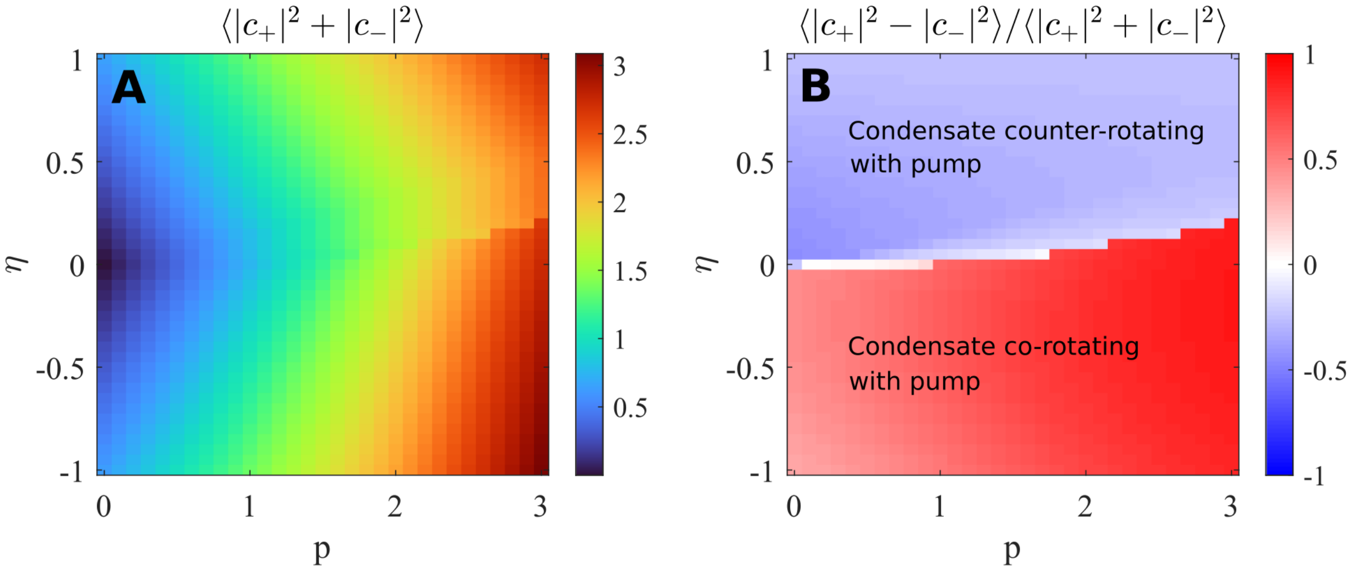

Figure S3. **Results of the numerical simulation of the Reduced Gross-Pitevskii equation.A** Time-averaged intensity and **B** vorticity of the condensate by numerically solving Eq. (S1) as a function of power p and dissipative coupling strength η. Other parameters are $\tilde{\alpha} = 1$ and $\Delta\omega = 1$ (pump rotating counterclockwise).
